# Vitamin C Deficiency Reduces Muscarinic Receptor Coronary Artery Vasoconstriction and Plasma Tetrahydrobiopterin Concentration in Guinea Pigs

**DOI:** 10.3390/nu9070691

**Published:** 2017-07-03

**Authors:** Gry Freja Skovsted, Pernille Tveden-Nyborg, Maiken Marie Lindblad, Stine Normann Hansen, Jens Lykkesfeldt

**Affiliations:** Department of Veterinary and Animal Sciences, Faculty of Health and Medical Sciences, University of Copenhagen, Ridebanevej 9, 1870 Frederiksberg C, Denmark; ptn@sund.ku.dk (P.T.-N.); mali@sund.ku.dk (M.M.L.); snoha@sund.ku.dk (S.N.H.); jopl@sund.ku.dk (J.L.)

**Keywords:** Vitamin C, ascorbic acid, vascular responses, biopterins

## Abstract

Vitamin C (vitC) deficiency is associated with increased cardiovascular disease risk, but its specific interplay with arteriolar function is unclear. This study investigates the effect of vitC deficiency in guinea pigs on plasma biopterin status and the vasomotor responses in coronary arteries exposed to vasoconstrictor/-dilator agents. Dunkin Hartley female guinea pigs (*n* = 32) were randomized to high (1500 mg/kg diet) or low (0 to 50 mg/kg diet) vitC for 10–12 weeks. At euthanasia, coronary artery segments were dissected and mounted in a wire-myograph. Vasomotor responses to potassium, carbachol, sodium nitroprusside (SNP), U46619, sarafotoxin 6c (S6c) and endothelin-1 (ET-1) were recorded. Plasma vitC and tetrahydrobiopterin were measured by HPLC. Plasma vitC status reflected the diets with deficient animals displaying reduced tetrahydrobiopterin. Vasoconstrictor responses to carbachol were significantly decreased in vitC deficient coronary arteries independent of their general vasoconstrictor/vasodilator capacity (*p* < 0.001). Moreover, in vitC deficient animals, carbachol-induced vasodilator responses correlated with coronary artery diameter (*p* < 0.001). Inhibition of cyclooxygenases with indomethacin increased carbachol-induced vasoconstriction, suggesting an augmented carbachol-induced release of vasodilator prostanoids. Atropine abolished carbachol-induced vasomotion, supporting a specific muscarinic receptor effect. Arterial responses to SNP, potassium, S6c, U46619 and ET-1 were unaffected by vitC status. The study shows that vitC deficiency decreases tetrahydrobiopterin concentrations and muscarinic receptor mediated contraction in coronary arteries. This attenuated vasoconstrictor response may be linked to altered production of vasoactive arachidonic acid metabolites and reduced muscarinic receptor expression/signaling.

## 1. Introduction

The association between vitamin C (vitC) deficiency and an increased risk of cardiovascular disease (CVD) in humans is well-established [1,2,3,4,5,6], though a mechanistic link is yet to be elucidated. VitC is an essential nutrient for humans and an estimated 10–15% of adults in the Western populations suffer from hypovitaminosis C (plasma concentration <23 μM) [7]. Pathologies of vascular diseases are characterized by changed vasomotion as a consequence of an imbalance in vasodilation and vasoconstriction, leading to abnormal blood flow regulation and organ dysfunction. In the vasculature, vitC is a key component in maintaining collagen integrity of blood vessels; prolonged severe deficiency ultimately leads to the development of bleedings and haematoma hallmarking scurvy. However, little is known about how non-scorbutic vitC deficiency affects the function of the vascular cells and the vasomotion of arterioles proximal to the capillaries.

In human endothelial cells in vitro, vitC has been shown to act as specific redox modulator in the nitric oxide (NO) synthesis essential for vasodilation [8]. Furthermore, vitC provides reducing equivalents in the conversion of dihydrobiopterin (BH_2_) to tetrahydrobiopterin (BH_4_) [9,10], which in turn acts as co-factor for endothelial nitric oxide syntase (eNOS) to ensure the generation of NO [11]. Reduced BH_4_ levels lead to eNOS uncoupling, and generation of superoxide rather than NO [12], likely to form the strong oxidant peroxynitrite and decreasing NO bioavailability [9]. We have previously shown that vitC deficiency in guinea pigs leads to decreased BH_4_ plasma concentration in vivo [13], potentially weakening the vasodilator capacity.

Another important aspect of vitC is its antioxidant function, serving as a scavenger of reactive oxygen species (ROS) [14]. ROS play key roles in signal transduction related to both vasodilation and vasoconstiction [15] and can inhibit the two major endothelium-derived relaxing factors, NO and prostacyclin [15]. While eNOS uncoupling reduces NO bioavailability, peroxynitrite can inactivate prostacyclin synthase by tyrosin nitration of its active site [16,17]. Thus, as the primary vascular antioxidant, vitC protects the endothelium from oxidative stress [18].

A third mechanism of vitC-associated action could be on the cholinergic response of vascular smooth muscle cells (VSMCs). Increased oxidative stress has been found to reduce muscarinic receptor function in smooth muscle cells of the urinary bladder [19], and studies have shown an attenuated parasympathetic response [20] and muscarinic-cholinergic receptor density [21] in submandibular gland acinar cells in vitC deficient guinea pigs. As coronary arteries express muscarinic receptors in the endothelium and VSMCs, and an extensive network of cholinergic perivascular nerve fibres in the coronary artery tree is present in guinea pigs [22], the acetylcholine analogue carbachol was applied to study both vasodilator and vasoconstrictor responses.

In contrast to rats and mice, guinea pigs and humans share a requirement for dietary vitC. This makes the guinea pig an excellent in vivo model for studying effects of diet-imposed vitC deficiency. In this study, we examined a causal relationship between chronic vitC deficiency and plasma biopterin redox status, and putative consequences on the vasodilator and vasoconstrictor responses in isolated coronary arteries.

## 2. Materials and Methods

### 2.1. Animal Study

All experimental animal procedures were performed following protocols approved by the Danish Animal Experimental Inspectorate under the Ministry of Environment and Food of Denmark (No. 2012-15-2934-00205). Group sizes were determined by power analysis of sample size, applying a power of 80% and a 5% significance level. A difference of 30% was considered biologically relevant and variations of the chosen end-points were based on our previous experience with the model [23,24]. Animals were selected by randomization from a larger, extensive, study of diet imposed vitC deficiency in guinea pigs intended to investigate vitC transport to the brain (unpublished results). Thus, the current data-set depicts findings from a randomly defined subset of animals, representing high vitC intake (control) and low (deficient), thereby, reflecting the extremes of the imposed interventions of the main study.

Seven-day old, Dunkin Hartley female guinea pigs (Envigo, Horst, The Netherlands) were equipped with subcutaneous microchip implant for identification (Uno Pico Transponder, Zevenaar, The Netherlands) upon arrival to the facility. Animals were randomized into weight stratified groups and subjected to either high (*n* = 16; 1500 mg vitC/kg feed; Controls) or low vitC (*n* = 16, 0 mg vitC/kg feed for 3 weeks, followed by 50 mg vitC/kg feed until study termination; Deficient). All diets were chow based standard guinea pig diets for growing animals (Ssniff Spezialdiäten, Soesst, Germany), differing only in vitC levels as confirmed by post production analysis. Animals were group-housed in identical floor pens and allowed free access to feed, dried hay (devoid of vitC by analysis) and drinking water. Body-weight was monitored throughout the study period, and though vitC deficient animals experienced a brief period (1–3 days) of weight stagnation immediately prior to changing from 0 mg to 50 mg vitC/kg feed, clinical signs of vitC deficiency were absent and body weight was comparable between groups at the time of euthanasia, 10–12 weeks after study start.

### 2.2. Euthanasia

Guinea pigs were sedated with Torbugesic Vet (2 mL/kg) (Butorphanol 10 mg/mL; ScanVet Animal Health, Fredernsborg, Denmark) and anesthetized with 5% isofluorane (Isoba Vet 100%, Intervet International, Boxmeer, The Netherlands) in oxygen (Conoxia^®^ 100%, AGA A/S, Copenhagen, Denmark) until cessation of voluntary reflexes. Blood was collected by cardiac puncture through the apex using a 18 G needle fitted onto a 1 mL syringe previously flushed with 15% tripotassium EDTA. Immediately hereafter, the guinea pig was euthanized by decapitation.

### 2.3. Wire Myography and Tissue Preparation

Immediately following euthanasia, the heart was isolated and placed into cold physiological buffer (in mM: 117.8 NaCl, 4.0 KCl, 2.0 CaCl_2_, 0.9 MgCl_2_, 1.25 NaH_2_PO_4_, 20 NaHCO_3_, and 5.0 glucose). The left anterior descending (LAD) coronary artery was dissected from surrounding myocardial tissue, cut into 2 mm segments and directly mounted in a wire myograph (Danish Myo Technology, Aarhus, Denmark). The anatomical localization of the LAD coronary artery is illustrated in Supplemental Figure S1. Wire myography experiments were initiated by normalisation to an internal circumference corresponding to 0.9 of the circumference at 13.3 kPa. Following a 15 min equilibration period in physiological buffer the artery segments were contracted 2–3 times using 60 mM potassium (similar composition as the above physiological buffer, except that NaCl was exchanged with KCl on equimolar basis) to measure the vasoconstrictor reactivity of the arteries. Only segments with potassium induced contraction >0.5 mN/mm were included in the study. After washing to obtain baseline relaxation, the ETB receptor agonist, Sarafotoxin 6c (S6c) was added in a cumulative fashion (10^−12^ to 10^−7^ M). Carbachol induced vasodilation and vasoconstriction (10^−12^ to 3 × 10^−4^ M) was tested following pre-constriction with potassium (40 mM). In order to elucidate the carbachol vasomotor responses, carbachol concentration-response curves were acquired either in absence (controls) or in presence of the muscarinic receptor antagonist, atropine (10^−5^ M), the COX-inhibitor indomethacin (10^−4^ M) or the eNOS inhibitor L-NAME (10^−5^ M). Endothelium-independent vasodilation was tested by sodium nitroprusside (10^−9^ to 10^−5^ M) following pre-constriction with 40 mM potassium. U46619 (10^−12^ to 10^−5^ M) and endothelin-1 (ET-1)-induced (10^−12^ to 10^−7^ M) vasoconstriction were tested using cumulative additions.

### 2.4. Biochemical Analysis

EDTA-stabilized blood samples were centrifuged (16,000× *g*, 1 min, 4 °C). Plasma for ascorbate and dehydroascorbic acid (DHA) analysis was stabilized with meta-phosphoric acid prior to storage at −80 °C. Previous studies have shown that both ascorbate and DHA are stable under these conditions for at least five years [23]. Concentrations were measured by HPLC with colorimetric detection as previously described [25,26]. The remaining plasma aliquots were stored neat at −80 °C until further analysis, except for samples for BH_2_ and BH_4_ determination, where the blood was stabilized in 0.1% dithioerythritol prior to centrifugation (2000× *g*, 4 min, 4 °C), yielding a plasma fraction, which was analyzed by HPLC, as previously described [13].

### 2.5. Data and Statistical Analysis

Force data (mN) were transformed to tension (Nm^−1^) by dividing by twice the artery segment length and subtracting the baseline tension values [24]. Active tension was calculated by subtracting the passive tension from the potassium-induced active tension. Agonist-induced tension was normalized to the potassium induced active tension. Carachol-induced relaxation was calculated by subtracting the active tensions from the potassium-induced (40 mM) active tension. All statistical analysis and graphs were performed in GraphPad Prism 7.00 (GraphPad Software, La Jolla, CA, USA). Differences between two groups were evaluated by a two-sided Student’s *t*-test. For multiple comparisons (in functional myography data), two-way ANOVA repeated-measures with Sidak’s multiple comparisons was applied. Correlations between specific outcomes were evaluated by Pearson correlation (r) coefficient with two-tailed *p*-values.

### 2.6. Materials

Endothelin-1, human, porcine (Catalogue No. SC324, PolyPeptide Group, Strassbourg, France), Sarafotoxin S6c (SC457, PolyPeptide Group, Strassbourg, France), 9,11-dideoxy-11α,9α-epoxy- methanoprostaglandin F2α (BML-PG023-0001, Enzo Life Sciences, Exeter, UK). Carbamoylcholine chloride (C4382-1G SIGMA, Sigma-Aldrich, St. Louis, MO, USA), indomethacin (I7378-5G SIGMA, Sigma-Aldrich, St. Louis, MO, USA), Nω-nitro-l-arginine methylester hydrochloride (N5751 Sigma, Sigma-Aldrich, St. Louis, MO, USA), atropine (A0132 SIGMA, Sigma-Aldrich, St. Louis, MO, USA), meta-phosphoric acid (239275, Sigma, Sigma-Aldrich, St. Louis, MO, USA), 1,4-dithioerythritol (D9680, Sigma, Sigma-Aldrich, St. Louis, MO, USA).

## 3. Results

### 3.1. Effects of Vitamin C Deficiency on Weight Gain of Animals, Plasma Ascorbate Concentration and Plasma BH_4_ Concentration

**Figure 1:**
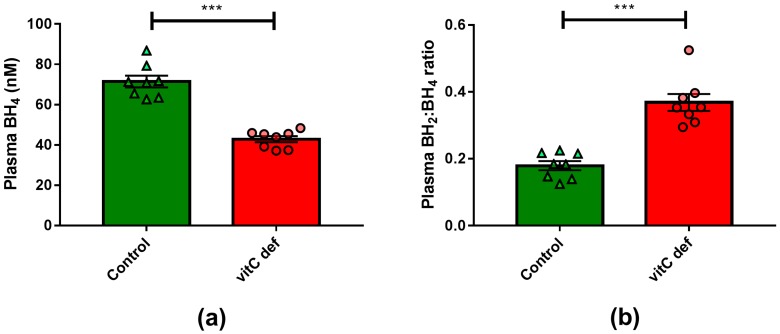
(**a**) Plasma concentrations of BH_4_; (**b**) plasma BH_2_:BH_4_-ratio. Means ± SEM, *** *p* < 0.0001 (*n* = 8).

Plasma vitC concentrations were measured at euthanasia, following 11 weeks on the experimental diets. The dietary regimen was reflected in plasma ascorbate and DHA concentrations, with marked (*p* < 0.001) reduction in plasma ascorbate concentration in the deficient group compared to the control group (Table 1). VitC deficiency also led to a significant reduction in plasma BH_4_ concentration (*p* < 0.0001) (Figure 1).

### 3.2. Contractile Reactivity

**Figure 2:**
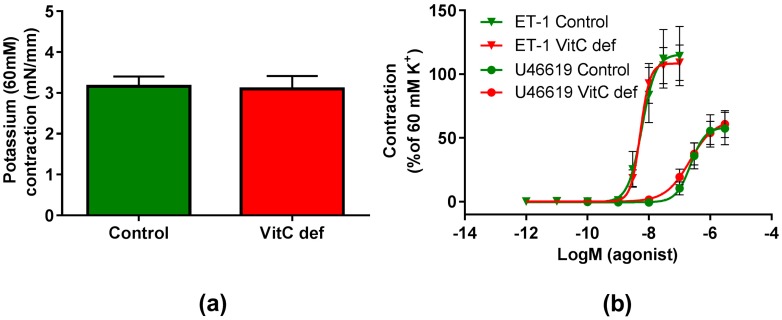
Contractile responses in coronary arteries. (**a**) Contractile responses to 60 mM extracellular potassium; (**b**) contractile responses to cumulative concentrations of ET-1 and U46610 in coronary arteries from control guinea pigs (green) and vitC deficient (red). Means ± SEM (K^+^, *n* = 16; ET-1, *n* = 12; U46619, *n* = 16).

**Figure 3:**
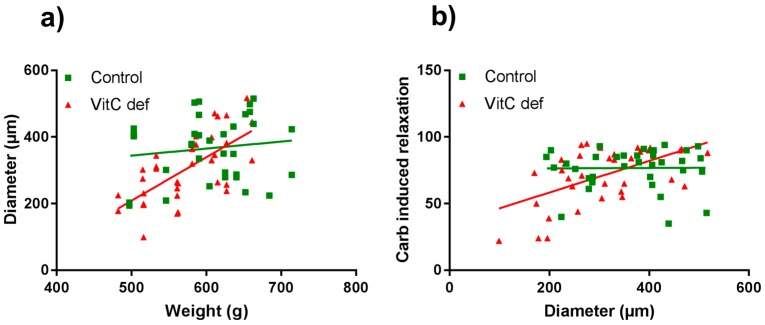
Scatter plots of coronary artery diameter vs. guinea pig body weight (**a**) and carbachol-induced vasorelaxation compared with coronary artery diameter (**b**). Control guinea pigs (green squares) and VitC deficient (red triangles).

The potency and efficacy of ET-1 was significantly higher than that of U46619 (*p* < 0.05), and the selective ETB receptor agonist, S6c, induced only a negligible contraction in the coronary artery segments (Table 2). VitC status did not have a significant effect on the potassium, ET-1, U46619 or S6c vasoconstrictor responses (Figure 2a,b). In contrast to the other vasoconstrictors, potassium induced a long-lasting vasocontractile response persisting for at least 10 min and potassium was therefore used as a pre-constrictor in the studies of the relaxation-inducing agonists. Coronary arteries from vitC deficient guinea pigs were significantly smaller than the controls (*p* < 0.05, Table 2). Additionally, we found that for the vitC deficient group, the animal weight was positively correlated with the coronary artery diameter (*p* < 0.002, Table 3 and Figure 3a).

### 3.3. Vascular Responses to Carbachol

**Figure 4:**
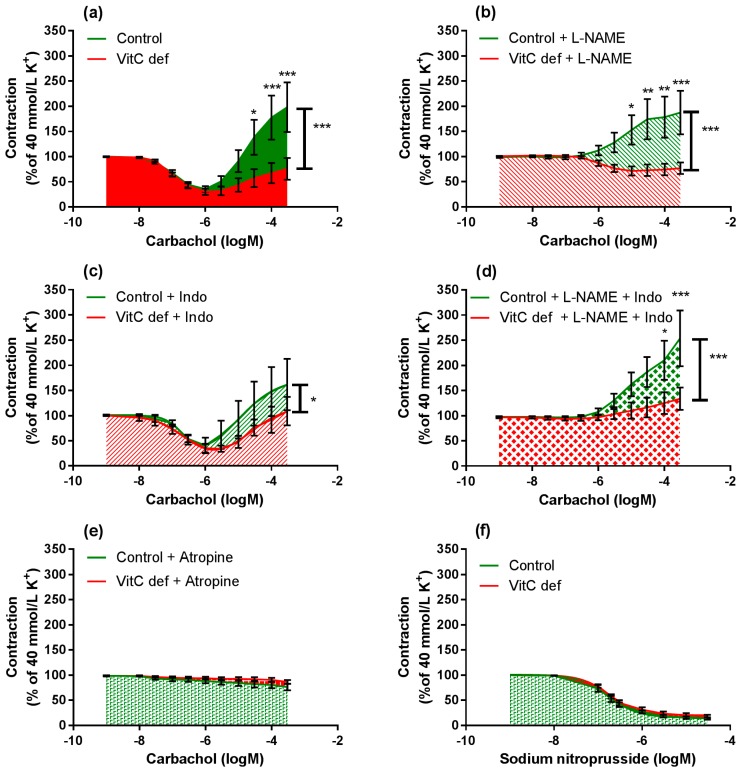
Log-concentration-response curves of coronary artery segments from vitC versus control guinea pigs. (**a**) Vasomotor responses to carbachol in coronary artery segments pre-constricted with 40 mM extracellular potassium; (**b**) in presence of L-NAME; (**c**) indomethacin; (**d**) both L-NAME and indomethacin; (**e**) atropine. (**f**) Vasodilator responses to sodium nitroprusside (SNP) in coronary arteries pre-constricted with 40 mM extracellular potassium. Control guinea pigs (green) and VitC deficient (red). Means ± SEM (*n* = 8–16), * *p* < 0.05, ** *p* < 0.01, *** *p* < 0.001.

Cumulative concentrations of carbachol in the range of 1 nM to 1 µM markedly relaxed coronary artery segments pre-contracted with 40 mM potassium (Figure 4a) and higher concentrations (1 µM to 0.3 mM) caused a rise in the isometric tension in a concentration-dependent fashion. Carbachol-induced relaxation and sensitivity was not significantly different in coronary arteries from vitC deficient guinea pigs compared to controls (Figure 3a); however, a significantly positive correlation between coronary artery diameter and carbachol-induced relaxation was found in coronary arteries from vitC deficient guinea pigs, but not in controls (*p* < 0.001), Table 3 and Figure 3b). The maximal carbachol-induced contraction response was significantly lower in segments from vitC deficient compared to controls (*p* < 0.001; Figure 4a) and in both vitC deficient and control animals the contractions were independent on coronary artery diameter (*p* > 0.05, Table 3). The muscarinic receptor antagonist, atropine (10 µM), blocked both the carbachol-induced vasodilation and vasoconstriction from both diet groups (Figure 2b). To evaluate the contribution of NO and prostanoids to carbachol-induced vasodilation and vasoconstriction, carbachol concentration- response curves were recorded during pre-contraction induced by potassium and in the presence of the COX inhibitor, indomethacin (10 µM), NOS inhibitor, L-NAME (100 µM) or in the presence of both indomethacin (10 µM) and L-NAME (100 µM). The presences of inhibitors alone or in combination revealed no differences in the vasodilator responses in control compared with vitC deficient animals, and the augmented vasoconstrictor responses in arteries from control animals compared to segments from vitC deficient animals were maintained in the presence of the inhibitors (Figure 4b–d).

**Figure 5:**
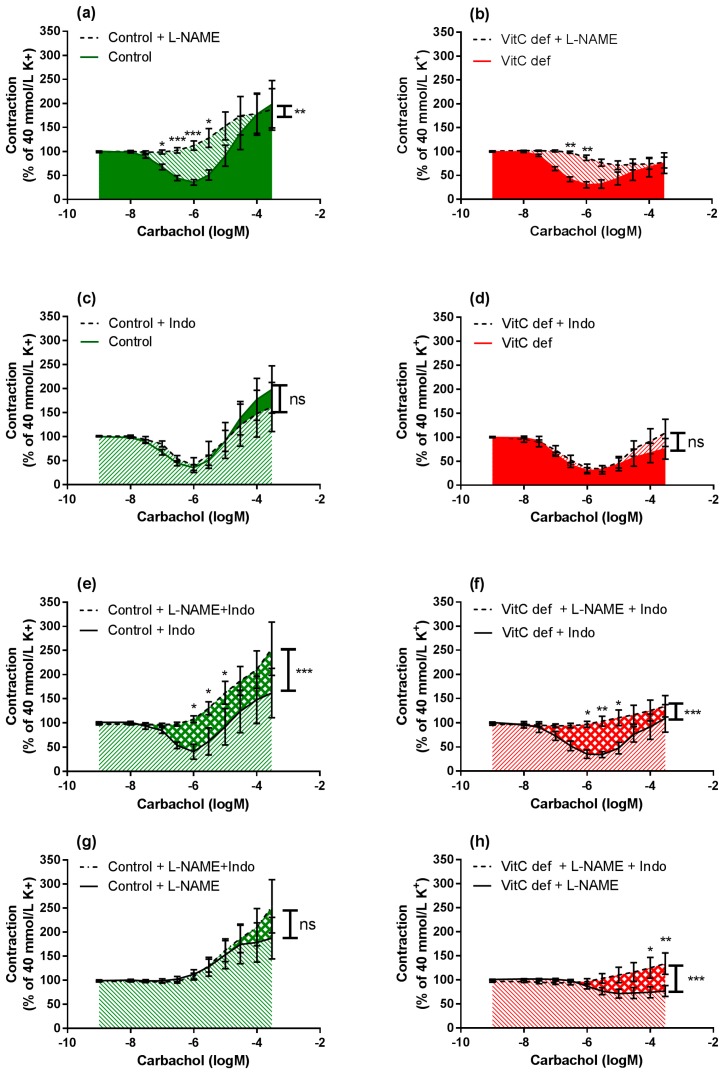
Log-concentration-response curves of coronary artery segments after treatment with L-NAME and/or indomethacin. The figures illustrate how the combination of inhibitors in artery segments from the same animal modulates the carbachol-induced vasodilator and constrictor responses. Vasomotor responses to carbachol in artery segments pre-constricted with 40 mM extracellular potassium: in absence versus presence of L-NAME in (**a**) control guinea pigs; (**b**) vitC deficient. In absence versus presence of indomethacin in (**c**) control; and (**d**) vitC deficient, in absence of L-NAME versus presence both L-NAME and indomethacin in (**e**) control guinea pigs; (**f**) VitC deficient, and in absence of indomethacin versus presence both L-NAME and indomethacin in (**g**) control guinea pigs; (**h**) VitC deficient. Means ± SEM (*n* = 8–16), * *p* < 0.05, ** *p* < 0.01, *** *p* < 0.001.

L-NAME significantly inhibited the vasodilatory response to carbachol in segments from both control and vitC deficient guinea pigs (*p* < 0.001; Figure 5a,b); however L-NAME alone had no effect on the subsequent vasoconstrictor response compared to non-treated segments. In the presence of indomethacin alone, both carbachol-induced vasodilatation and vasoconstriction were restored (Figure 5c,d) in arteries from both groups compared to non-treated segments.

In the presence of L-NAME, indomethacin amplified the carbachol-induced vasoconstriction only in segments from vitC deficient guinea pigs, suggesting a potential effect arachidonic acid metabolites e.g., vasodilator prostanoids counteracting the vasoconstrictor effect of carbachol in coronary arteries from vitC deficient guinea pigs or increased production of vasoconstrictor leukotrienes, which is unmasked in the presence of indomethacine. This effect was not recorded for control animals.

In summary, we found that carbachol-induced vasodilator and constrictor responses were mediated by muscarinic receptors. In vitC deficient guinea pigs, the diameter of the coronary arteries were significantly and positively correlated with the weight of the animals and the endothelium-dependent vasorelaxation. These correlations were not present in the control group. In vitC deficient guinea pigs the muscarinic receptor-induced vasoconstrictor responses were significantly attenuated compared to controls and partly restored by COX-inhibition.

### 3.4. Relaxing Responses to SNP

To evaluate the endothelium-independent response to NO, relaxing response to the NO donor SNP (1 nM to 30 µM) was measured in potassium pre-contracted arteries. SNP induced a concentration dependent relaxation and the maximal relaxation and the sensitivity to SNP were not affected by the vitC status in the animals (Figure 4f).

## 4. Discussion

The present study shows that vasoconstrictor responses to carbachol are significantly decreased in arteries from vitC deficient guinea pigs as compared to arteries from control animals, proposing a link between vitC deficiency and compromised vascular function in vivo. Interestingly, contractions induced by other constrictors: potassium, S6c, U46619 and ET-1 were not affected by vitC status, suggesting that the contractile apparatus *per se* is not affected. Although vitC deficiency decreased plasma BH_4_ levels, there was no significantly decreased vasodilator capacity compared to controls. However, vitC deficient guinea pigs had significantly smaller coronary artery diameters than controls, and in vitC deficient guinea pigs, the decreased diameter correlated with decreased carbachol-induced vasodilatation. Consequently, it appears that impaired vitC status affects the diameter of the coronary arteries and the endothelial function; furthermore vitC status induces a specific effect on the parasympathetic muscarinic receptor system, as measured by attenuated vasoconstrictor responses.

The parasympathetic neurotransmitter, acetylcholine and its analogue carbachol are widely used to study endothelial dependent/independent vasodilation and vasoconstriction, and the agonist is relevant since guinea pigs have an extensive network of cholinergic perivascular nerve fibres in the coronary artery tree [22]. In isolated coronary artery segments, carbachol induced a biphasic concentration-response pattern with an initial vasodilator response at low concentrations (from 10 nM to 1 µM) followed by a vasoconstrictor response at higher concentrations (from 1 µM to 0.3 mM). Carbachol-responses in the presence of atropine, indomethacin and/or L-NAME in the organ bath were assessed, revealing that atropine blocked both the carbachol-induced vasodilatation and vasoconstriction over the entire carbachol concentration interval. This suggests that carbachol elicits its effect via muscarinic receptors on guinea pig coronary arteries. This is consistent with previous studies showing that acetylcholine-induced vasodilator responses in bovine [25], simian [26] and mice [27] coronary arteries are mediated predominantly by endothelial muscarinic M_3_ receptors, and that acetylcholine induced vasoconstrictor responses are mediated by vascular M_3_ receptors in bovine [28,29] and porcine [30,31] coronary arteries.

In this study, we found that in vitC deficient guinea pigs, the endothelial-dependent vasodilation was significantly correlated with coronary artery diameter; a correlation that was not present in controls. Furthermore, we found coronary artery diameters were significantly smaller in vitC deficient guinea pigs as compared to control guinea pigs, despite sampling at uniform, anatomically defined, orientation. These results suggest that vitC deficiency potentially impair coronary artery growth and endothelial function of young guinea pigs. Hence, those guinea pigs that responded most sensitively to vitC restriction further developed more overall growth retardation with consequently impaired coronary artery growth and endothelial function. In the control group, in contrast, the variation in artery diameter did not reflect a pathophysiological response, but rather, a random variation in growth. Previously, degenerative changes in the capillary endothelium have been found in scorbutic guinea pigs whereas the larger arteries showed no abnormalities [32]. However, although we found this correlation between artery diameter and endothelial function in vitC deficient animals and not in controls; we found no overall effect of vitC deficiency when comparing the two groups. Treatment of the coronary artery segments with L-NAME abolished the initial carbachol-induced vasodilator response in both diet groups. In contrast, indomethacin did not significantly affect the vasodilator response between groups, suggesting that carbachol-induced vasodilator response was predominantly driven by endothelial-dependent NO release. Importantly, the vasodilator responses were investigated in arteries preconstricted with high extracellular concentration of potassium. High potassium concentrations depolarize VSMC and endothelial cells [33] which consequently hide a putative endothelium-derived hyperpolarizing factor (EDHF) mediated vasodilator effect [34]. Therefore, blocking the contribution of EDHF to vasodilation allowed the isolation of the effects of NO and prostaglandins on carbachol induced vasodilation.

Muscarinic receptors are widely expressed in smooth muscle cells in several organs, and diet-induced ascorbate deficiency in guinea pigs has previously been shown to reduce muscarinic-cholinergic receptor density [21]. Increased oxidative stress has been found to acutely reduce muscarinic receptor-mediated smooth muscle cell constriction in guinea pigs [19], and increased ROS production in ischemia/reperfusion reduce efficacy and sensitivity to cholinergic stimulation [35,36], linking redox imbalance to functional consequences mediated via muscarinic receptors.

Stimulating the arteries with the NO donor sodium nitroprussid revealed vasodilation with similar sensitivity and maximal effect in arteries from vitC deficient and control guinea pigs (Figure 4f). NO mediates a vasodilator effect by binding to soluble guanylyl cyclase (sGC) in VSMC. Guanylyl then catalyses the production of cGMP, which activates protein kinase G that via dephosphorylation of myosin light chain leads to vasorelaxation [37]. Oxidative stress has been showed to down-regulate soluble guanylyl cyclase expression and activity [38]. In present study, we found no effect of vitC deficiency on the NO-mediated vasodilation, indicating that the sGC activity was unaltered by the vitC status.

When coronary artery segments were treated with L-NAME and/or indomethacin, the carbachol-induced vasoconstrictor response remained reduced in arteries from vitC deficient compared to control animals. However, indomethacin increased the carbachol-induced vasoconstrictor response in eNOS blocked segments from vitC deficient animals, which was not present in arteries from control animals. This suggests that vitC deficiency promotes the release of vasodilator prostanoids in coronary arteries when stimulated with carbachol. Vasodilator prostanoids have previously been found to negate the effect of coronary vasoconstrictors after myocardial infarction [39], a condition known to induce oxidative stress [40] and be detrimental to cellular function and survival. In contrast, inhibition of prostanoid production has been found to have little effect in healthy humans [41] and dogs [42]. In this study, prostanoid-induced suppression of carbachol vasoconstrictor responses in coronary arteries were increased in vitC deficient guinea pigs, suggesting a compensatory role in the regulation of coronary vascular tone under vitC and BH_4_ deficiency. The contractile responses induced by either extracellular potassium, U46619, S6c or ET-1 were not affected by vitC status, supporting the idea that the general vasoconstrictor capacity is not affected by vitC deficiency.

VitC deficiency (defined as plasma concentrations <23 μM) is surprisingly common, affecting ~15% of adults in the Western World with even higher prevalence among individuals who smoke, have high BMI, low socioeconomic status [7] as well as children with underlying medical conditions [43,44]. Epidemiological studies have shown an association between vitC deficiency and an increased risk of cardiovascular disease; however, the mechanistic link has not been elucidated [45,46]. Altered vasomotion and reactivity of coronary arteries plays an important role in pathophysiologic mechanisms involved in heart disease. The parasympathetic nervous system is known to provide a modulating influence on the response of coronary arteries to local metabolic requirements in the heart [47]. Moreover, muscarinic receptors are known to be expressed in human coronary arteries [48]. Patients with variant angina or coronary stenosis have been found to have altered coronary vasoconstriction after injection of acetylcholine, pointing toward a causal relationship with altered muscarinic receptor response and disease [49,50]. The observation that muscarinic receptor mediated contraction is impaired as a result of vitC deficiency is of potential importance, not only in regulation of coronary artery vasomotion, but also in other tissues that are highly dependent on parasympathetic-muscarinic receptor-mediated contraction (bladder, esophagus, intestines, pancreas, and salivary glands). For obvious ethical reasons, it is impossible to perform long-term controlled trials on humans to establish the consequence of vitC deficiency on vasculature and present knowledge is therefore restricted to indirect evidence. Applying the guinea pig as a unique and validated model of diet-induced vitC deficiency, this study shows that chronic vitC deficiency in vivo alters the response of the coronary arteries to parasympathetic stimuli. This provides a link between vitC deficiency and cardiovascular disease, proposing a yet undisclosed, specific, effect of vitC in the modulation of muscarinic receptor-modulated response within the vascular wall. Though requiring further investigation, the apparent association between vitC status and coronary artery contraction may prove relevant in the prevention and treatment of cardiovascular diseases in humans with poor vitC status.

The present study has several limitations. Based on existing literature, we expected that vitC deficiency reduced NO-mediated vasodilation as a consequence of the decreased BH_4_ plasma concentration. The potential reason for the lack of correlation between the BH_4_ plasma concentrations and endothelial function could be that we determined biopterines and vitC in the plasma, rather than in the arteries, which could potentially more adequately have reflected the vessel status. Interestingly, a correlation between animal weight, artery diameter and endothelial function in vitC deficient guinea pigs was found. Future studies are needed to elaborate on a causal relationship and putative functional consequences e.g., clarifying if vitC deficiency induces morphological changes of the heart muscle and vessels. Furthermore, measurements of intracellular calcium concentrations would be highly relevant to determine if vitC deficiency alters cytosolic calcium levels and handling, which can lead to increased tone and decreased vessel diameter. Here, the effect of vitC deficiency was evaluated in young guinea pigs—reproductive maturity is reached at around 10 weeks of age—with no other underlying pathophysiological condition. We found that the coronary arteries were highly resistant to mechanically endothelial denudation, suggesting that the animals, despite vitC deficiency, retained a high NO capacity and/or sensitivity in the coronary arteries. However, it could be speculated that in the presence of an additional vascular disease, such as atherosclerosis, left ventricular hypertension [51] or even age-related reductions in compensatory abilities, a decreased vitC concentration and consequently, a reduced capacity to recycle BH_4_, may be crucial in preserving an adequate vasodilator capacity [52].

Our finding, that the muscarinic receptor system is highly sensitive to vitC deficiency, is a novel and so far unrecognized effect of in vivo vitC deficiency. Future studies are needed to elucidate the mechanisms underlying the impaired muscarinic receptor mediated contraction observed here and to study other tissues with highly dependent parasympathetic-muscarinic receptor-mediated contraction (e.g., bladder, esophagus, intestines, pancreas, and salivary glands).

## 5. Conclusions

The present study shows that chronic vitC deficiency impairs vasomotor function of coronary arteries. During vitC deficiency, the endothelial function is reduced, with decreasing vessel diameter and carbachol-induced vasoconstrictor responses being significantly impaired. The carbachol-induced effects are apparently mediated by altered muscarinic receptor activity. Although further studies are required to evaluate the underlying mechanisms and the potential clinical implications, these findings may provide a link between chronic vitC deficiency and increased risk of cardiovascular disease reported in numerous epidemiological studies.

## Figures and Tables

**Table 1:** Animal weight and plasma analyses. Data are expressed as means ± SEM, N is number of animals, **** Different from controls, *p* < 0.0001, unpaired *t*-test.

	N	Controls	N	VitC Deficient
Weight (g)	16	625 ± 14	16	602 ± 16
Ascorbate concentration, (µM)	16	60.2 ± 5.1	16	2.3 ± 0.1 ****
Ascorbate total, (µM)	16	61.5 ± 5.3	16	2.3 ± 0.1 ****
% DHA	16	1.9 ± 0.5	16	2.2 ± 1.0

**Table 2:** Artery segment properties, diameter (µm), potassium tension (Nm^−1^), agonist induced contraction (% of potassium contraction). Data are expressed as means ± SEM, N is number of animals, n is number of artery segments, NC = not calculated. * Different from controls, *p* < 0.05, unpaired *t*-test.

	N, n	Controls	N, n	VitC Deficient
Diameter	16, 35	366 ± 16	16, 35	312 ± 12 *
Potassium, tension	16, 35	3.2 ± 0.2	16, 35	3.1 ± 0.3
Endothelin-1	12, 17	Emax 116 ± 16	12, 17	Emax 108 ± 8
		pEC_50_ 8.2 ± 0.1		pEC_50_ 8.3 ± 0.1
Sarafotoxin 6c	8, 20	Emax NC	8, 20	Emax 3 ± 1.6
		pEC_50_ NC		pEC_50_ 8.5 ± 0.7
U46619	16, 35	Emax 58 ± 9	16, 35	Emax 64 ± 13
		pEC_50_ 6.6 ± 0.1		pEC_50_ 6.7 ± 0.2

**Table 3:** Correlation analyses of specific outcomes in the control and vitC deficient groups. Pearson correlation analyses, N is number of animals, n is number of artery segments, r is Pearson correlation coefficient, ** *p* <0.002, *** *p* < 0.001.

	Controls			VitC Deficient		
	N, n	Pearson r	*p* Values	N, n	Pearson r	*p* Values
Diameter vs. weight	16, 35	0.182	0.500	16, 35	0.729	0.001 **
Diameter vs. carb induced dilatation	16, 35	0.010	0.954	16, 35	0.555	0.0005 ***
Diameter vs. carb induced contraction	16, 35	−0.174	0.518	16, 35	−0.286	0.284
BH_4_ vs. carb induced dilatation	8, 15	0.403	0.105	8, 15	0.057	0.840
BH_4_ vs. carb induced contraction	8, 15	−0.372	0.172	8, 15	0.232	0.406

## Supplementary Materials

The following are available online at www.mdpi.com/2072-6643/9/7/691/s1; Figure S1: Localization of coronary artery segments used for myograph experiments.
