# Supplementary figures and images for: Vitamin C Deficiency Reduces Muscarinic Receptor Coronary Artery Vasoconstriction and Plasma Tetrahydrobiopterin Concentration in Guinea Pigs
Source: Nutrients. 2017 Jul 3;9(7):691. doi: 10.3390/nu9070691 (PMC5537806; doi:10.3390/nu9070691)

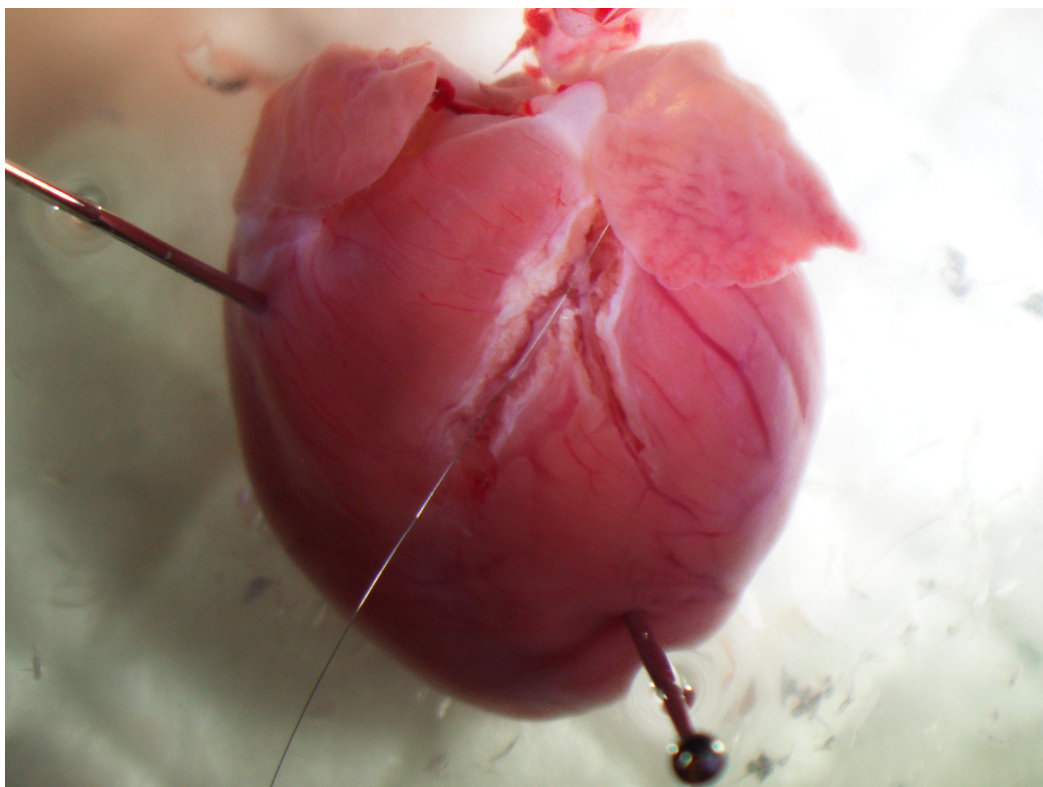

**Figure S1.** Localization of coronary artery segments used for myograph experiments.

Supplement: Supplementary file 1 [file nutrients-09-00691-s001.zip › nutrients-196982-supplementary.pdf]
